# Dysregulation of autophagy as a common mechanism in lysosomal storage diseases

**DOI:** 10.1042/EBC20170055

**Published:** 2017-12-12

**Authors:** Elena Seranova, Kyle J. Connolly, Malgorzata Zatyka, Tatiana R. Rosenstock, Timothy Barrett, Richard I. Tuxworth, Sovan Sarkar

**Affiliations:** 1 Institute of Cancer and Genomic Sciences, College of Medical and Dental Sciences, University of Birmingham, Birmingham B15 2TT, U.K.; 2 Department of Physiological Science, Santa Casa de São Paulo School of Medical Science, São Paulo, SP 01221-020, Brazil

**Keywords:** Autophagy, Glycogenoses, Lysosomes, Lysosomal storage disorders, Neuronal ceroid lipofuscinoses, Sphingolipidoses

## Abstract

The lysosome plays a pivotal role between catabolic and anabolic processes as the nexus for signalling pathways responsive to a variety of factors, such as growth, nutrient availability, energetic status and cellular stressors. Lysosomes are also the terminal degradative organelles for autophagy through which macromolecules and damaged cellular components and organelles are degraded. Autophagy acts as a cellular homeostatic pathway that is essential for organismal physiology. Decline in autophagy during ageing or in many diseases, including late-onset forms of neurodegeneration is considered a major contributing factor to the pathology. Multiple lines of evidence indicate that impairment in autophagy is also a central mechanism underlying several lysosomal storage disorders (LSDs). LSDs are a class of rare, inherited disorders whose histopathological hallmark is the accumulation of undegraded materials in the lysosomes due to abnormal lysosomal function. Inefficient degradative capability of the lysosomes has negative impact on the flux through the autophagic pathway, and therefore dysregulated autophagy in LSDs is emerging as a relevant disease mechanism. Pathology in the LSDs is generally early-onset, severe and life-limiting but current therapies are limited or absent; recognizing common autophagy defects in the LSDs raises new possibilities for therapy. In this review, we describe the mechanisms by which LSDs occur, focusing on perturbations in the autophagy pathway and present the latest data supporting the development of novel therapeutic approaches related to the modulation of autophagy.

## Introduction

Our perspective of the lysosome has shifted remarkably from its standing as a simple, terminal organelle for the degradation of cellular components to become a critical mediator of fundamental metabolic processes. Lysosomes coordinate signals from growth factors and cellular stressors and are sensitive to various metabolites, such as amino acids, glucose, lipids and cholesterol, to pivot cells between anabolic and catabolic processes, including autophagy [1–3]. The importance of the lysosome for cellular function is apparent from the large number of disorders associated with lysosomal failure: collectively known as the lysosomal storage disorders (LSDs), more than 50 inherited conditions affect lysosomal function and many are early-onset and fatal [4–7].

A key cellular homeostatic pathway implicated in several LSDs and myriad human diseases is autophagy. Autophagy is an intracellular degradation pathway essential for cellular survival and organismal health [8–10]. This process is vital for the maintenance of energy and tissue homeostasis by degrading damaged or excess intracellular components such as aggregation-prone proteins, lipids and organelles, and recycling the breakdown products [11]. There are three types of autophagy: macroautophagy (herein referred to as autophagy), microautophagy and chaperone-mediated autophagy (CMA); each type requires functional lysosomes in the final stage for degradation of the cargo [12]. Consequently, disruption of the hydrolytic functions of lysosomes impairs autophagic flux and, conversely, lysosomal function probably requires normal flux through autophagy [13]. Deregulation of autophagy is a common disease mechanism in many LSDs [14,15]. This review will describe the connections between autophagy and the LSDs, highlight common stages of autophagy disrupted in different disorders and discuss autophagy as a potential therapeutic intervention for treating certain LSDs.

## Autophagy machinery and signalling

**Figure 1:**
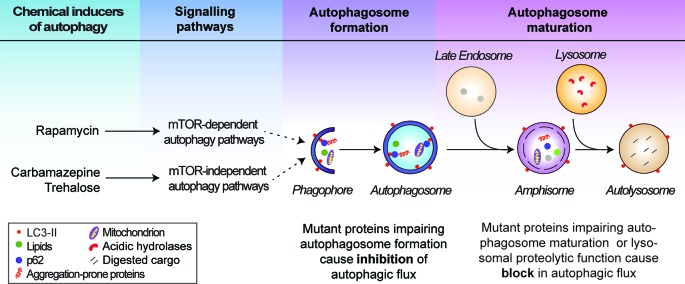
Schematic representation of the autophagy pathway Autophagy initiates by the *de novo* synthesis and elongation of phagophores, which engulf cytosolic materials (autophagic cargo) to form autophagosomes. Autophagosomes predominantly fuse with the late endosomes to form amphisomes and subsequently with the lysosomes to form autolysosomes where the autophagic cargo is degraded by the lysosomal hydrolases. Autophagy can be stimulated by chemical inducers acting via the mTOR-dependent and mTOR-independent pathways regulating autophagy. Defects in autophagic flux at the autophagosome formation and maturation stages are indicated.

Autophagy encompasses several vesicle fusion events leading to the eventual degradation of its cargo; a dynamic process termed autophagic flux (Figure 1). Multiple autophagy (*Atg*) genes encoding components of the autophagic machinery are required for the initiation of autophagy, which is marked by the *de novo* formation of double-membrane structures called phagophores. Two ubiquitin-like conjugation systems involving the Atg5–Atg12–Atg16 complex and phosphatidylethanolamine-conjugated microtubule‐associated protein 1 light chain 3 (LC3-II) are required for the initiation step [16,17]. Cytosolic components such as macromolecules (including specific substrates like p62) and organelles (including mitochondria) are sequestered in the expanding phagophores to form double-membrane vesicles called autophagosomes. LC3-II associates with autophagosome membranes throughout their lifespan and is hence used as a marker for autophagy [18]. Autophagosomes then undergo maturation into autolysosomes by one of two routes: the predominant route is a multi-step process, in which autophagosomes fuse first with late endosomes to form amphisomes, then subsequently with lysosomes; alternatively, autophagosomes can fuse directly with lysosomes [19,20]. This enables delivery of the autophagic cargo to the autolysosomes where these materials are degraded by acidic lysosomal hydrolases (Figure 1). The breakdown products are then exported via lysosomal transporters for recycling [21].

There are several molecular mediators of autophagosome maturation, including Rab7, Beclin1–Vps34–Vps15 complex and SNAREs (*N*-ethylmaleimide-sensitive factor-attachment protein receptors). Beclin1-interacting partners like Atg14L, Ambra1 and UVRAG promote autophagosome maturation whereas Rubicon inhibits this step [20,22,23]. Recently, gene knockout studies in mammalian cells have shown that the Atg8 family proteins (LC3 and GABARAP subfamilies) are crucial for autophagosome–lysosome fusion [24]. GABARAPs were found to preferentially recruit PLEKHM1 (Pleckstrin homology domain containing protein family member 1) [24], which associates with the homotypic fusion and protein sorting (HOPS) complex to mediate autophagosome maturation [25]. The SNARE proteins are critical for membrane tethering and fusion; for example, autophagosomal Syntaxin-17, Atg14 and SNAP-29 interact with late endosomal/lysosomal VAMP8 to mediate autophagosome maturation [26–28]. Upon autolysosome formation, the ATG conjugation system has been suggested to play a role in the degradation of the inner autophagosomal membrane [29]. The late stage of autophagy involving autophagosome maturation is primarily affected in many LSDs; the perturbations in the autophagy pathway in these conditions are described in subsequent sections (Table 1).

**Table 1:** Overview of defective autophagy in lysosomal storage disorders

Disease	Gene	Protein	Function	Storage material	Autophagy phenotype	Autophagic flux	Mechanism
**NEURONAL CEROID LIPOFUSCINOSES**							
CLN2	*CLN2/TPP1*	Tripeptidyl peptidase 1	Serine protease	ATPase subunit c, lipofuscin	Inhibition of autophagosome formation; Reduction in autophagosomes and autophagic degradation [60]	Inhibition	Up-regulation of mTOR signalling [60]
CLN3	*CLN3*	CLN3	Unknown function; Lysosomal membrane protein	ATPase subunit c, lipofuscin	Defect in autophagosome maturation; Accumulation of autophagosomes and autophagic cargo [58–60]	Block	Not known; Possibly due to alteration in Ca^2+^ homeostasis [61] and deregulation of ARF1–Cdc42 pathway [62]
CLN5	*CLN5*	CLN5	Unknown function; Lysosomal protein	ATPase subunit c, lipofuscin	Accumulation of autophagosomes and autophagic cargo [56]	Block	Not known
CLN6	*CLN6*	CLN6	Unknown function; ER membrane protein	ATPase subunit c, lipofuscin	Accumulation of autophagosomes and autophagic cargo [55,57]	Block	Not known
CLN7	*CLN7*	CLN7	Putative lysosomal transporter	ATPase subunit c, lipofuscin	Accumulation of autophagosomes and autophagic cargo [56]	Block	Not known; Possibly due to impairment in lysosomal function [54]
CLN10	*CLN10/CTSD*	Cathepsin D	Aspartyl protease	ATPase subunit c, saposins A/D, lipofuscin	Accumulation of autophagosomes and autophagic cargo [64,66]	Block	Not known; Possibly due to loss of cathepsin D function [64]
**SPHINGOLIPIDOSES**							
Niemann–Pick type C1	*NPC1*	NPC1	Cholesterol transporter	Unesterified cholesterol, sphingolipids	Defect in autophagosome maturation; Accumulation of autophagosomes and autophagic cargo [73–80]	Block	Disruption in SNARE machinery [73]; Reduction in sphingosine kinase activity and VEGF [80]
Niemann–Pick type C2	*NPC2*	NPC2	Putative role in cholesterol metabolism and transport	Unesterified cholesterol, sphingolipids	Accumulation of autophagosomes and autophagic cargo [84]	Block	Not known; Possibly due to impairment in lysosomal function [84]
Gaucher disease	*GBA1*	Glucocerebrosidase	Sphingolipid degradation	Glucosylceramide	Defect in autophagosome maturation; Accumulation of autophagosomes and autophagic cargo [91,93–97]	Block	Not known; Possibly due to down-regulation of TFEB and reduction in lysosomes [91]
	*PSAP*	Prosaposin, saposin C	Sphingolipid hydrolase cofactor	Glucosylceramide	Defect in autophagosome maturation; Accumulation of autophagosomes and autophagic cargo [92,93]	Block	Not known; Possibly due to reduction in cathepsin B/D activity [92]
Mucolipidosis type IV	*MCOLN1*	TRPML1	Late endo-lysosomal Ca^2+^ transporter	Gangliosides, phospholipids, mucopolysaccharides	Accumulation of autophagosomes and autophagic cargo [104,105,109]	Block	Not known; Possibly due to impairment in lysosomal function [107]
**GLYCOGENOSES**							
Pompe disease	*GAA*	Acid α-glucosidase	Glycogen degradation	Glycogen	Accumulation of autophagosomes and autophagic cargo [116–118]	Block	Not known; Possibly due to defects in lysosomal acidification [116]
Danon disease	*LAMP2*	Isoform LAMP2b	Putative role in autophagosome–lysosome fusion	Glycogen	Accumulation of autophagosomes and autophagic cargo [123–126]	Block	Not known; Possibly due to defects in lysosomal function [124]
X-linked myopathy with excessive autophagy	*VMA21*	VMA21	Regulates v-ATPase	Glycogen	Accumulation of autophagosomes [128,129]	Block	Not known; Possibly due to defects in lysosomal acidification and function [128]

The list in Table 1 highlights selected LSDs where defective autophagy has been demonstrated.

Transcriptional regulation of autophagy occurs via the transcription factor EB (TFEB), which drives the expression of genes related to autophagy and lysosomal biogenesis [30,31]. In turn, Ca^2+^ stored in the lysosomal lumen has been shown to modulate autophagy by promoting the nuclear localization of TFEB through the activation of calcineurin [32]. Various signalling pathways influence autophagy by acting upstream of the autophagic machinery. These include the mechanistic target of rapamycin complex 1 (mTORC1) signalling pathway (through which growth factors, nutrients and energy status influence autophagy) and mTOR-independent pathways (including inositol and IP_3_, cAMP, Ca^2+^, calpain) that negatively regulate autophagy [12,33–38]. Both mTOR-dependent and mTOR-independent signalling are amenable to chemical perturbations for modulating autophagy [34,37,39–41] (Figure 1). For instance, the mTORC1 inhibitors like rapamycin and torin1, and mTOR-independent compounds including trehalose and carbamazepine can stimulate autophagy [35,42–44]. Chemical inducers of autophagy have been tested in some LSDs as a possible therapeutic intervention (Table 2) and are described below.

**Table 2:** Beneficial effects of the chemical inducers of autophagy in models of lysosomal storage disorders

Autophagy inducer	Mechanism of autophagy induction	LSD	Beneficial effects in LSD models
**mTOR-DEPENDENT AUTOPHAGY INDUCER**			
Rapamycin [42]	Inhibition of mTORC1 [42]	NPC1	Rescue of autophagic flux and improvement in cell viability in mutant *Npc1* MEFs [73], mouse neurons with *Npc1* knockdown [73] and NPC1 patient iPSC-derived neuronal and hepatic cells [74]
		NPB	Reduction in mitochondrial ROS and lipid droplets, and induction of lysosomal exocytosis in NPB patient B lymphocytes [148]
		PD	Reduction in muscle glycogen in *Gaa*-deficient mice when treated together with recombinant human GAA [167]; Improved autophagic flux and GAA maturation in Pompe disease patient myotubes [117]
		GD	Improvement in lifespan and locomotor activity in GD *Drosophila* model [97]
**mTOR-INDEPENDENT AUTOPHAGY INDUCER**			
Trehalose [44]	Inhibition of SLC2A glucose transporters [145]; Activation of TFEB by Akt inhibition [144]	NPC1	Rescue of autophagic flux and improvement in cell viability in NPC1 patient iPSC-derived neurons [74]
		CLN3	Clearance of ceroid lipopigment deposits in CLN3 patient fibroblasts, and attenuation of neuropathology and extension of lifespan in *Cln3*-deficient mice [144]
Carbamazepine [35]	Reduction in inositol and IP_3_ levels [35]	NPC1	Rescue of autophagic flux and improvement in cell viability in NPC1 patient iPSC-derived neurons and hepatic cells [74]
Lithium [35]	Inhibition of IMPase and reduction in inositol and IP_3_ levels [35]	NPC1	Rescue of autophagic flux in mutant *Npc1* MEFs [73]
		CLN3	Rescue of autophagic flux and improvement in cell viability in mutant *Cln3* cerebellar cells [146]
L-690,330 [35]	Inhibition of IMPase and reduction in inositol and IP_3_ levels [35]	CLN3	Rescue of autophagic flux and improvement in cell viability in mutant *Cln3* cerebellar cells [146]
Verapamil [36]	Inhibition of L-type Ca^2+^ channel and reduction in cytosolic Ca^2+^ [36]	NPC1	Rescue of autophagic flux and improvement in cell viability in NPC1 patient iPSC-derived neurons [74]
BRD2716, BRD5631, BRD34009 [142]	Unknown	NPC1	Rescue of autophagic flux and improvement in cell viability in NPC1 patient iPSC-derived neurons [142]

Abbreviations: CLN, ceroid lipofuscinosis neuronal disease; GD, Gaucher disease; IMPase, inositol monophosphatase; IP3, inositol 1,4,5-trisphosphate; iPSC, induced pluripotent stem cells; LSD, lysosomal storage disorder; mTORC1, mechanistic target of rapamycin complex I; NPB, Niemann–Pick type B disease; NPC1, Niemann–Pick type C1 disease; PD, Pompe disease; SLC2A, Solute carrier 2A; TFEB, Transcription factor EB.

## Lysosomal storage diseases associated with defective autophagy

LSDs are caused by abnormal lysosomal function leading to accumulation of undegraded metabolites [4–7]. The composition of accumulated materials in the lysosomes varies substantially between the LSDs and, while all LSDs are inherited conditions and in many cases mutations are found in genes encoding lysosomal proteins, the types of proteins affected are also varied. As a consequence, the underlying cell biology changes occurring in the LSDs may vary, but each results (directly or indirectly) in reduced clearance of aggregates and diminished cellular homeostasis and survival. Autophagic dysregulation is commonly found in LSDs but again, a spectrum of defects is seen at various stages of the autophagic pathway in different LSDs (Table 1) [14,15]. Here, we will concentrate on some of the better-studied LSDs and detail the different autophagic defects identified.

## Neuronal ceroid lipofuscinosis

**Figure 2:**
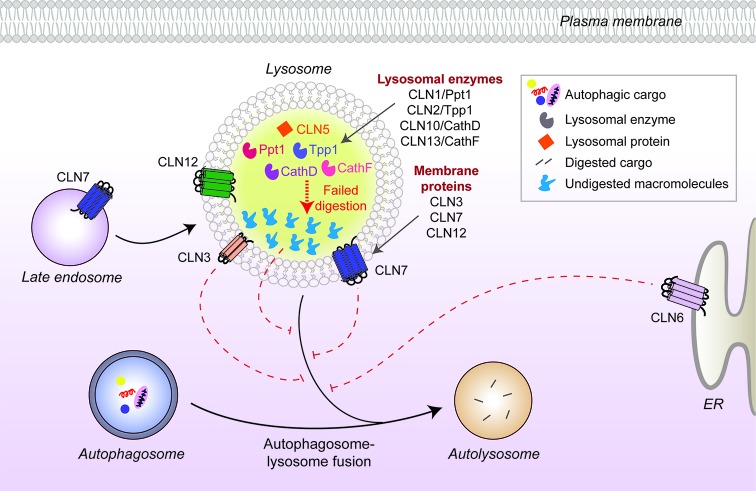
CLN protein distribution and their link to autophagy defects in neuronal ceroid lipofuscinoses Many CLN proteins reside in the lysosomal matrix (CLN1, 2, 5, 10, 13) or at the lysosomal membrane (CLN3, 7), while others localize to different cellular compartments such as the ER membrane (CLN6). Disease-causing mutations in some of the CLN proteins inhibit autophagosome maturation (dashed red lines) and block autophagic flux, but the underlying mechanisms are unknown. Mutated lysosomal hydrolases (CLN1, 2, 10, 13) are unable to degrade autophagic cargo, which subsequently accumulate and impair lysosomal function.

While defective autophagy has been implicated in the pathology of many different disorders, including cancer and various cardiovascular, metabolic, pulmonary and infectious diseases [10,45], the nervous system appears to be particularly susceptible [46,47]. This is likely due to a combination of the long-lived nature of post-mitotic neurons placing particular stress on protein-clearing processes, the extreme polarization of many neurons and the high metabolic requirements of neurons leading to higher levels of oxidative damage in lysosomes via the Fenton reaction. Consistent with this, dysregulated autophagy has been identified in almost every late-onset neurodegenerative disorder [34,47,48], and similar defects are now being recognized in the neuronal ceroid lipofuscinoses (NCLs), a sub-group of the LSDs that are collectively the most common causes of childhood-onset neurodegeneration [5,49,50]. To date, 13 disease-causing genes have been identified to cause NCL pathology. Several of these genes encode lysosomal proteins, including soluble enzymes/proteins (CLN1/PPT1, CLN2/TPP1, CLN5, CLN10/CTSD and CLN13/CTSF) and membrane proteins (CLN3, CLN7/MFSD8 and CLN12/ATP13A2), but others include endoplasmic reticulum (ER) membrane proteins (CLN6 and CLN8), cytosolic proteins (CLN4/DNAJC5 and CLN14/KCTD7) and one expressed in the secretory pathway (CLN11/GRN) [51–53]. The diverse nature of these proteins, their substrates or cargos and locations within the cell are reflective of the LSDs as a whole: multiple different cell biological processes are probably affected but with each terminating in common pathology. However, as autophagic perturbations start to be identified in NCL disease models (Figure 2), the possibility that each disease hinges on defective autophagy becomes more plausible.

Several studies in NCL mouse models have indicated deregulation of autophagy *in vivo*. A recent study in *Cln7*-deficient mice reported accumulation of autophagosomes and autophagic substrates coupled with lysosomal dysfunction in the brain, suggesting that a block in autophagic flux may be occurring [54]. A similar build-up of autophagic compartments and substrates was also seen in mouse models of CLN5 and CLN6 diseases, as well as in CLN6 patient-derived fibroblasts [55–57]. The most common form of NCL is Batten disease, caused by mutations in the membrane protein, CLN3 [51]. Defective autophagosome maturation associated with increased autophagic and lysosomal compartments was observed in the *Cln3*-deficient mouse that models Batten disease, and also in patient fibroblasts as well as patient-specific induced pluripotent stem cell (iPSC)-derived neuronal cells [58–60]. The mechanism underpinning the changes to autophagy in CLN3-deficient cells is not fully understood, and the functions of the CLN3 protein remain unknown, but one potential mechanism may be due to altered Ca^2+^ homeostasis and deregulation of the ARF1–Cdc42 pathway identified in *CLN3* mutant cells that can impact on vesicular trafficking [61,62].

Several forms of NCL are caused by mutations in lysosomal proteases, including Ppt1 (CLN1), Ppt2 (CLN2) and Cathepsin D (CLN10) [53,63]. Loss of cathepsin function commonly leads to dysregulated autophagy, presumably by reducing autophagic flux due to defective cargo clearance. Autophagosomes and mitochondrial proteins accumulate in cultured neurons of mice lacking cathepsin D or B and L [64–66], while *in vivo*, cathepsin D-deficient mice exhibit widespread accumulation of storage material in the lysosomes (indicating lysosomal dysfunction) and accumulation of autophagosomes. These changes likely lead to the neurodegeneration present in these mice that mimics the human disease [66]. However, a reduction in autophagic flux has been reported in CLN2 patient fibroblasts due to inhibition of autophagosome formation, which is linked to increased ROS and Akt–mTOR signalling pathway [60].

## Sphingolipidoses

Sphingolipids are a major class of lipids enriched in the nervous system and are critical for neural development and function [67,68]. Sphingolipid turnover is therefore tightly regulated with their degradation mediated by a multi-step process requiring numerous lysosomal hydrolases [69]. The sphingolipidoses are a class of LSDs caused by deficiencies in functional hydrolases, culminating in the accumulation of wholly or partially undegraded sphingolipids [68]. Members of the sphingolipidoses include Niemann–Pick, Gaucher and Fabry diseases, mucolipidosis, and GM1/2 gangliosidoses such as Tay-Sachs and Sandhoff diseases [68]. Alterations in autophagy have been reported in some of the sphingolipidoses; the most extensively studied being Niemann–Pick type C1 (NPC1) disease.

### Niemann–Pick disease

Niemann–Pick disease is a recessively inherited neurodegenerative condition characterized by an accumulation of unesterified cholesterol deposits in various tissues, particularly the brain. The disease is subdivided into types A, B, C1 and C2. Types A and B are caused by mutations in *SMPD1*, leading to loss of acid sphingomyelinase activity, whereas types C1 and C2 are due to defective cholesterol transport [70]. Ninety-five per cent of cases are attributed to mutations in the lysosomal cholesterol transporter NPC1, with the lysosomal glycoprotein NPC2 that possibly aids in cholesterol trafficking affected in the remainder [71,72].

**Figure 3:**
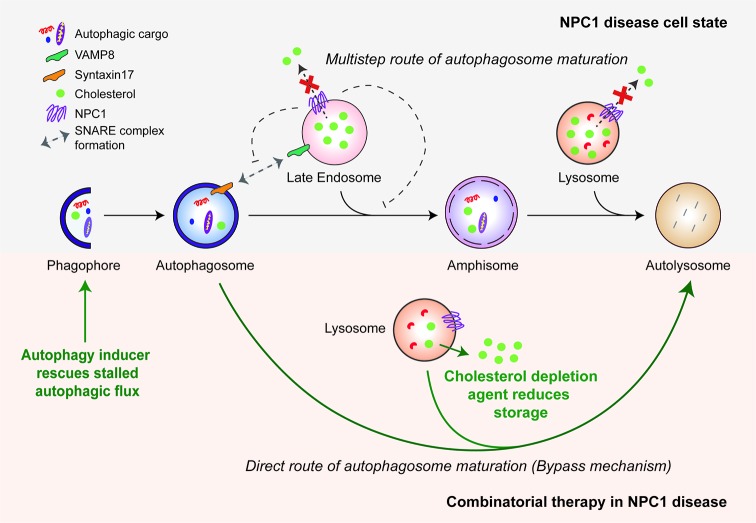
Autophagy defects in NPC1 disease and the bypass mechanism of autophagosome maturation for restoring autophagic flux Mutant NPC1 protein prevents cholesterol efflux from the endo-lysosomal compartments and impairs autophagosome maturation in the multi-step route due to failure in the SNARE machinery. Induction of autophagy by chemical inducers bypasses this block and restores autophagic flux via direct autophagosome–lysosome fusion. A combinatorial treatment strategy is shown with cholesterol depletion agents. The green arrows indicate therapeutic effects of autophagy induction and cholesterol depletion.

A number of studies have demonstrated a major role for autophagy in the pathology of NPC1 disease and potentially in treatment options. Various experimental platforms, including *Npc1* mutant mice and disease-relevant cells (such as neurons) differentiated from human embryonic stem cells (hESCs) with *NPC1* knockdown or patient-derived iPSCs, have revealed an accumulation of autophagosomes and lysosomes both *in vivo* and *in vitro* [73–80]. We previously reported that this autophagy phenotype is related to a block in autophagic flux (Figure 3). Consistent with this, we characterized a failure in the Syntaxin17/VAMP8 SNARE machinery that retards amphisome formation and significantly stalls the multi-step route of autophagosome maturation [73]. While it is possible that loss-of-function of the NPC1 protein, which normally resides on the late endosomal/lysosomal compartments, directly perturbs amphisome formation, accumulation of lysosomal cholesterol has also been reported to cause aberrant sequestration of SNAREs and prevent autophagosome maturation [81]. Another study, although consistent with dysfunctional autophagosome maturation, reported a distinct mechanism in which diminished sphingosine kinase activity and reduced levels of vascular endothelial growth factor (VEGF) lead to the accumulation of sphingosine that, in turn, impairs autophagic flux [80]. In addition to these mechanisms, depletion of lysosomal Ca^2+^ stores due to sphingosine storage was seen in NPC1 disease models, and this could possibly influence autophagy via calcineurin or calpain [32,36,82,83]. Similar to the autophagy phenotype in NPC1 disease, a block in autophagic flux has been reported in NPC2 disease as evident by the accumulation of autophagosomes and autophagic substrates as well as impaired lysosomal activity in *Npc2*-knockdown mouse adipocytes [84].

The reduced cell viability seen in NPC1 mouse and patient-specific iPSC models has been attributed to dysregulated autophagy [73,74,78,80]. Importantly, since autophagy regulates the clearance of lipids including cholesterol (via lipophagy), autophagy-deficient *Atg5^–/–^* cells also exhibited accumulation of these materials similar to the cellular phenotypes observed in NPC1 disease [73,85]. Therefore, the underlying autophagy defects in NPC1 disease can further aggravate the accumulation of lipids and cholesterol, and thus, defective autophagy should be considered a major disease mechanism in Niemann–Pick disease.

### Gaucher disease

Gaucher disease is the most common form of sphingolipidosis [86], and is caused by a deficiency in glucocerebrosidase (GCase) activity which catalyses the final step in glycosphingolipid degradation [87]. The loss of enzyme function in Gaucher disease directly impairs this process leading to accumulation of glucocerebroside and widespread pathology that particularly affects the liver and, in severe cases, the CNS [88]. Gaucher disease is most frequently caused by mutations in the *GBA1* gene which encodes GCase [87]; however, there are rare cases of Gaucher disease caused instead by mutations in the *PSAP* gene [89,90]. *PSAP* encodes the lysosomal glycoprotein prosaposin, the precursor saposins A-D, which are essential cofactors for various sphingolipid hydrolases. Saposin C is required for GCase activity, and thus mutations in *PSAP* that lead to saposin C deficiency also cause Gaucher disease [89,90].

Various lines of evidence suggest defective autophagy in Gaucher disease. Impaired autophagosome maturation and down-regulation of TFEB, including a reduction in lysosomal gene expression, were found in neurons differentiated from patient-specific iPSCs [91]. A similar impairment in autophagosome degradation was also seen in primary fibroblasts deficient in saposin C; in this case associated with reduced cathepsin B/D activity [92]. Mouse models of Gaucher disease have also revealed similar autophagy phenotypes: accumulation of various autophagic cargo such as dysfunctional mitochondria, ubiquitinated protein aggregates, insoluble α-synuclein and p62, together with autophagosomes and lysosomes were found in the brain or neurons and astrocytes cultured from mice deficient for *Gba, Psap* or glucosylceramidase [93–96]. Recently, a *Drosophila* model of neuropathic Gaucher disease generated by knocking out the *Gba* gene revealed severe lysosomal defects, GCase accumulation and a block of autophagic flux in the brain, resulting in reduced lifespan, neurodegeneration and age-dependent locomotor deficits [97]. Together, multiple model systems highlight deregulation of autophagy in Gaucher disease.

### Mucolipidosis type IV

**Figure 4:**
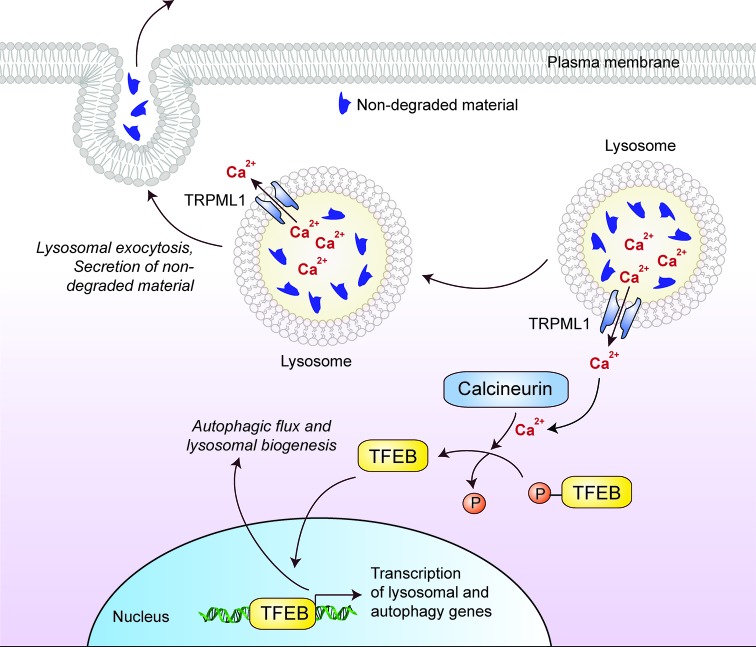
Cellular effects of TFEB that might be of therapeutic benefit in lysosomal storage disorders Lysosomal Ca^2+^ efflux through TRPML1 activates the Ca^2+^-dependent phosphatase calcineurin, which mediates dephosphorylation-dependent nuclear translocation of TFEB. Nuclear TFEB up-regulates the transcription of genes involved in lysosome biogenesis and autophagy, thereby enhancing autophagic flux. In addition, Ca^2+^ efflux from peripheral lysosomes promotes lysosomal exocytosis and the secretion of non-degraded materials.

Mucolipidosis type IV (MLIV) is a neurodegenerative condition, caused by mutations in the *MCOLN1* gene that result in the loss of TRPML1 function [98,99]. TRPML1 is an inward rectifying, non-selective cation channel of the transient receptor potential family that transports divalent cations, such as Ca^2+^, Fe^2+^ and Zn^2+^, from the lysosomal intraluminal space into the cytosol [100–102]. TRPML1 and TFEB positively regulate each other and together have the potential to impact on autophagy: Ca^2+^ release by TRPML1 activates the Ca^2+^-dependent phosphatase, Calcineurin, which mediates dephosphorylation-dependent nuclear translocation of TFEB leading, in turn, to increased expression of several lysosomal and autophagy-related proteins including TRPML1 [32] (Figure 4). Indeed, enhancing TRPML1 activity either by overexpression or by pharmacological stimulation increases autophagic flux [103].

Several models of MLIV have highlighted a defect in autophagy. Accumulation of lysosomes, autophagosomes and autophagy substrates including polyubiquitinated protein aggregates was reported in patient-derived primary fibroblasts, in *Mcoln1*-deficient mice, and in *Caenorhabditis elegans* and *Drosophila* models [104–108]. These studies also implicated a role for wild-type TRPML1 in regulating lysosomal acidification and autophagosome maturation (alkalinization of lysosomes, for instance by inhibiting the vacuolar H^+^ATPase, inhibits hydrolase function). Furthermore, defects in CMA have been reported in MLIV, possibly through the interaction between TRPML1 with the molecular chaperones Hsc70 and Hsc40 that are required for protein translocation to the lysosome during CMA [109].

## Glycogenoses

The glycogenoses are LSDs with profound autophagy defects that particularly affect skeletal and cardiac muscle [110,111]. As such, these diseases are often referred to as autophagic vacuolar myopathies (AVMs), although neuropathology is also present in some cases [112]. Diseases that lead to AVM are linked to genes involved in lysosome acidification, glycogen hydrolysis, and autophagosome maturation and fusion with lysosomes [113]. They include Pompe disease, Danon disease and X-linked myopathy with excessive autophagy (XMEA).

### Pompe disease

Pompe disease is caused by mutations in acid α-glucosidase (GAA), a lysosomal glycogen hydrolase [114]. Enzyme activity is completely absent in Pompe disease and consequently the accumulation of intra-lysosomal glycogen, as well as of autophagosomes, is a hallmark of the disease [115]. Primary myoblasts from *Gaa*-deficient mice have enlarged endosomes, lysosomes and autophagic vacuoles, in addition to delayed endosomal acidification and mobility [116]. These features have been attributed to a block in autophagic flux since elevated levels of the autophagosomal marker LC3-II and autophagic substrate p62 were observed in *Gaa*-deficient mice and in the myotubes of infantile-onset patients [117,118]. Interestingly, autophagy has been shown to play a role in the maturation of GAA [117] and the clearance of glycogen [119,120], and thus, defective autophagy is implicated as a disease mechanism in Pompe disease.

### Danon disease

Danon disease is an X-linked disorder caused by mutations in the *LAMP2* gene, which encodes a lysosomal membrane protein [121]. While LAMP2a is required for protein translocation in CMA [122], mutations in the LAMP2b isoform are associated with Danon disease [121]. Various tissues including liver, skeletal and heart muscle from *Lamp2*-deficient mice displayed accumulation of autophagic compartments, along with a decline in the lysosomal degradation of long-lived proteins in the hepatocytes that are unresponsive to amino acid starvation [123,124]. Muscle biopsies from patients also exhibit accumulation of LC3-II^+^ autophagic vesicles and large p62 aggregates [125]. These studies point to a likely block in flux affecting the late stage of autophagy. Consistent with this scenario, impaired autophagy associated with defective mitochondrial clearance (mitophagy) was found in cardiomyocytes differentiated from patient-specific iPSC cells with *LAMP2* mutations as well as in *Lamp2*-deficient mice [126].

### X-linked myopathy with excessive autophagy

X-linked myopathy with excessive autophagy (XMEA) is characterized by progressive vacuolation and atrophy of skeletal muscle, and is caused by mutations in the *VMA21* gene [127]. The VMA21 protein regulates the assembly of the vacuolar ATPase (v-ATPase) required for lysosomal acidification, and thus XMEA is associated with a reduction in the activity of lysosomal hydrolases [128]. Skeletal muscle biopsies show diminished lysosomal degradation and accumulation of autophagic compartments, suggesting a likely block in autophagic flux [128,129].

## Why is defective autophagy relevant for LSD pathology?

Deregulation of autophagy has been observed in myriad human diseases including many forms of cancer and neurodegeneration [10,45,47]. Despite appearing at first glance to have diverse changes in both the cell biology and the genetics underpinning them, deregulated autophagy has been reported in most (or possibly all) LSDs where investigated (Table 1) [14,15]. Potentially, dysfunctional autophagy is the common link between the LSDs but why is impaired autophagy such an important component of disease and how does it contribute to cell death? Clues have emerged from *in* vivo experiments aimed at depleting basal autophagy in a tissue-specific manner in mice. These result in degeneration and dysfunction of the targeted organ, indicating an essential role for autophagy in maintaining tissue homeostasis [130]. For example, brain-specific deletion of *Atg5* or *Atg7* in mice results in a neurodegenerative phenotype in the absence of any disease-causing, aggregation-prone proteins [131,132]. These genetic studies suggest that the decline in autophagic capability in the LSDs is likely a major contributory factor to the neuropathology that characterizes many of these disorders. Secondly, some of the non-degraded materials in LSDs such as lipids, cholesterol and glycogen are cleared by autophagy [85,119,120]. Hence, deficient autophagy will contribute to the build-up of these components with ever increasing impact on lysosomal function and autophagic flux. For example, as discussed in NPC1 disease where lipids and cholesterol are the primary non-degraded materials, impairment in autophagy will lead to a further accumulation of these macromolecules by reducing lipophagy [73]. This is because lipophagy is retarded due to loss of autophagy, as evident in autophagy-deficient mouse models [85,133]. Finally, impairment in functional autophagic flux can retard the clearance of autophagic cargo including damaged mitochondria and other undesirable materials, which leads to enhanced oxidative damage of lipids and proteins within the lysosomal membrane and a further reduction in efficiency of clearance [134–137]. Therefore, targeting this common pathway underlying several LSDs could be a promising therapeutic intervention.

## Autophagy as a potential therapeutic intervention for LSDs

Autophagy has been exploited for therapeutic benefits in diverse transgenic models of human diseases, including several neurodegenerative disorders, certain liver diseases, myopathies and infectious diseases [34,40,41,138,139]. In most of the conditions studied, induction of autophagy ameliorated the disease phenotypes and, in many cases, improved organismal longevity [140,141]. Multiple lines of evidence suggest that autophagy could also be targeted in LSDs as a treatment strategy [14,15] (Table 2). The most suitable disease contexts for this approach are likely to be LSDs where the lysosomal hydrolytic function is not overtly compromized (e.g., by direct mutation of an enzyme) so that the accumulated autophagic cargo can still be digested efficiently if autophagic flux is stimulated. Some of the mechanisms of how autophagy might be beneficial in LSDs are discussed below.

### Bypassing stalled autophagosome maturation by small-molecule autophagy inducers

The dysregulated autophagy underpinning many of the LSDs occurs primarily due to failure of autophagosome maturation, which stalls autophagic flux (Table 1). This phenotype probably arises due to a block in the multi-step route of autophagosome maturation. However, this block may not be absolute because we have recently characterized a bypass mechanism in NPC1 disease models which restores functional autophagic flux [73]. We have shown that induction of autophagy facilitates direct autophagosome–lysosome fusion without requiring the formation of amphisomes in the multi-step route (Figure 3). Restoration of autophagic flux enables cargo clearance, which correlates with improved cell viability in primary mouse neurons with *Npc1* knockdown and in NPC1 patient iPSC-derived neuronal and hepatic cells [73,74]. Interestingly, we observed cell-type specificity of small molecule autophagy enhancers in these patient-derived cells: rapamycin and carbamazepine rescued cell death and defective autophagy in both iPSC-derived neuronal and hepatic cells but other autophagy inducers including trehalose, verapamil, BRD2716, BRD5631 and BRD34009 were effective only in neurons [74,142]. Strikingly, enhanced autophagic flux did not reduce the cholesterol load in NPC1 mutant cells, meaning a combination strategy coupling autophagy induction with low doses of a cholesterol-depletion agent such as 2-hydroxypropyl-β-cyclodexterin (HPβCD) may be especially effective for NPC1 disease [73,74,143].

Treatment with autophagy inducers has also been found to be beneficial in other LSDs, including CLN3 disease (Batten disease). Administration of trehalose, an mTOR-independent autophagy inducer [44], in a mouse model of CLN3 disease promoted cellular clearance of undegraded materials, attenuated the neuropathology and extended lifespan [144]. Interestingly, although the autophagy-inducing property of trehalose has been recently attributed to the inhibition of the SLC2A family of glucose transporters [145], trehalose was found to activate TFEB by inhibiting Akt independently of mTORC1 [144]. Furthermore, inositol monophosphatase (IMPase) inhibitors such as lithium and L-690,330, which induce mTOR-independent autophagy by reducing inositol and IP_3_ levels [35], rescued autophagic flux and improved cell viability in mutant *Cln3* cerebellar cells [146]. However, further studies are required to determine whether the bypass mechanism of restoring autophagic flux is mediating the beneficial effects of autophagy induction in these contexts.

Induction of autophagy via the classical mTORC1 pathway also had some beneficial effects in LSD models. Although TORC1 activity is dysregulated in a *Drosophila* model of Gaucher disease [97], the mTORC1 inhibitor rapamycin [42] partially corrects the shortened lifespan and locomotor defects [97], whereas reactivation of dysregulated mTORC1 by knockdown of the Rheb inhibitor, TSC2, in a mouse model of Pompe disease rescued the muscular atrophy and autophagy defects [147]. Rapamycin, while commonly used in research contexts, particular in cell-based assays *in vitro*, is unlikely to be useful clinically in all LSDs, in part because its efficacy appears to be context-dependent *in vivo* and subject to feedback loops. Even *in vitro* rapamycin has differential effects. For example, it reduces mitochondrial ROS and lipid droplets and induces lysosomal exocytosis in Niemann–Pick type B lymphocytes [148], and improves autophagic flux and GAA maturation in Pompe disease patient myotubes [117], but is toxic in neuronal cells differentiated from Gaucher disease patient-derived iPSCs [91]. Since mTOR governs critical cellular function like cell growth and translation [38], the use of mTOR inhibitors for induction of autophagy can produce potential side-effects [149]. Consequently, the mTOR-independent autophagy inducers are considered more desirable for clinical applications in patients [34,40,41,138].

### TFEB promotes lysosomal biogenesis and exocytosis

Genetic up-regulation of autophagy via overexpression of TFEB has shown potential as a therapeutic strategy. Overexpression of TFEB enhances the clearance of proteinaceous aggregates in transgenic models of late-onset neurodegeneration disorders [150–152], and improves autophagy and lysosomal defects while rescuing disease severity in some LSDs [153,154]. Apart from mediating the transcription of genes required for autophagy and lysosomal biogenesis [30,31], TFEB also facilitates lysosomal exocytosis via the recruitment and fusion of the lysosomes to the plasma membrane by increasing local Ca^2+^ concentrations through its target, the TRPML1/MCOLN1 cation channel (Figure 4) [32,155]. This mechanism may underpin some of the beneficial effects of overexpression because enhanced secretion may enable the clearance of pathologically enlarged lysosomes and undegraded materials associated with LSDs that have an impact on autophagic flux.

Experimentally, overexpression of TFEB reduces glycogen load and the size of lysosomes, and promotes autophagosome maturation and lysosomal exocytosis in immortalized myogenic cells from Pompe disease patients [156] and rescues tissue pathology *in vivo* in autophagy-dependent manner [155,156]. In addition, overexpression of TFEB together with GAA cooperatively facilitated glycogen clearance and improved pathology in skeletal muscles differentiated from Pompe disease patient-derived iPSCs [157]. Similarly, lysosomal exocytosis and reduction in cellular vacuolization occur after TFEB overexpression in various cell and mouse models of LSDs, including multiple sulfatase deficiency (MSD) and mucopolysaccharidosis type IIIA (MPS-IIIA) where a block in autophagy was previously reported [155,158]. Overexpression of TFEB also causes reduction in substrate accumulation in various cell models of Pompe disease, MSD, MPS-II, MPS-IIIA and Batten disease [150,155,156], although it did not rescue the lysosomal depletion phenotype in neuronal cells differentiated from Gaucher disease patient-derived iPSCs [91]. However, TFEB did enhance lysosomal biogenesis in the presence of recombinant GCase in these cells [91]. Taken together, these studies highlight TFEB as a promising therapeutic target with the potential to be manipulated in multiple LSDs.

### Combining ERT with autophagy induction

Enzyme replacement therapy (ERT) to replace the absent or non-functional hydrolytic enzyme is approved for patients with Gaucher, Pompe and Fabry diseases and some mucopolysaccharidoses, including MPS type I [159,160]. For Gaucher disease, ERT for the GCase enzyme is a relatively mature therapy with several drugs on the market, including imiglucerase, velaglucerase alfa and taliglucerase alfa [161–163], along with two substrate reduction therapies, miglustat and eliglustat [164,165]. Recent data demonstrate that ERT for the GAA enzyme deficient in Pompe disease improves muscle mass and autophagic flux in muscle biopsies of some patients after treatment [117,166]. As with the Gaucher disease experiments described above [91], combination strategies that include induction of autophagy may prove more effective in Pompe disease and similar LSDs than ERT alone, in this case possibly by stimulating clearance of the accumulating glycogen and facilitating GAA maturation [117,157]. Indeed, experiments with a Pompe mouse model *in vivo* demonstrated increasing autophagic flux with rapamycin or its analogue CCI-779 alongside ERT for the GAA enzyme reduced muscle glycogen levels compared with either treatment alone [167]. These emerging data highlight the potential benefits of combinatorial treatment strategies in the LSDs.

## Conclusions

The LSDs are characterized by accumulation of undegraded macromolecules due to defective or absent lysosomal hydrolases, or because of dysregulated endosomal–lysosomal processes, including altered vesicular trafficking, autophagosome maturation, lysosomal acidification or transport of molecules across the lysosomal membrane. The separate classes of LSDs represent several different defects in lysosomal biology and to some extent require tailored therapies to overcome them. However, one feature apparent in many of the LSDs is dysregulated autophagy (Table 1), and this may be the common therapeutic target that can be exploited similarly in different disorders. Based on the common blockage of autophagic flux, therapies designed to stimulate autophagy and alleviate the block are a logical option to trial. While autophagy inducers have already shown benefit in a few LSDs (Table 2), a broader evaluation of autophagy modulation in the LSDs is urgently required. Autophagy induction shows particular promise in combination with existing therapeutic options such as ERT. However, ERT is applicable to only a subset of the LSDs and the treatment costs are high, and therefore, identifying novel therapeutic interventions for the LSDs that are cheaper and more widely useful is essential. Ultimately, different combinations of therapies are likely to be required for each of the LSDs but autophagy regulators may play a significant role in many.

## Summary

- Autophagy is a vital cellular process requiring the degradative function of lysosomes.
- Abnormal lysosomal function leading to accumulation of undegraded metabolites occurs in the LSDs.
- Defects in autophagy are emerging to be a common disease mechanism underlying LSDs.
- Stimulation of autophagy is a potential therapeutic intervention in LSDs.

## Abbreviations

ERT: enzyme replacement therapy
GAA: acid α-glucosidase
GCase: glucocerebrosidase
iPSC: induced pluripotent stem cells
LAMP2: lysosome-associated membrane protein 2
LSD: Lysosomal storage disorder
MLIV: mucolipidosis type IV
MPS: mucopolysaccharidosis
MSD: multiple sulfatase deficiency
mTORC1: mechanistic target of rapamycin complex 1
NCL: neuronal ceroid lipofuscinosis
NPC1: Niemann–Pick type C1 disease
ROS: reactive oxygen species
SNARE: *N*-ethylmaleimide-sensitive factor-attachment protein receptors
TFEB: transcription factor EB
XMEA: X-linked myopathy with excessive autophagy

## References

[1] Luzio J.P., Pryor P.R. and Bright N.A. (2007) Lysosomes: fusion and function. Nat. Rev. Mol. Cell Biol. 8, 622–632 17637737 10.1038/nrm2217

[2] Perera R.M. and Zoncu R. (2016) The lysosome as a regulatory hub. Annu. Rev. Cell Dev. Biol. 32, 223–253 27501449 10.1146/annurev-cellbio-111315-125125 PMC9345128

[3] Xu H. and Ren D. (2015) Lysosomal physiology. Annu. Rev. Physiol. 77, 57–80 25668017 10.1146/annurev-physiol-021014-071649 PMC4524569

[4] Futerman A.H. and van Meer G. (2004) The cell biology of lysosomal storage disorders. Nat. Rev. Mol. Cell Biol. 5, 554–565 15232573 10.1038/nrm1423

[5] Platt F.M., Boland B. and van der Spoel A.C. (2012) The cell biology of disease: lysosomal storage disorders: the cellular impact of lysosomal dysfunction. J. Cell Biol. 199, 723–734 23185029 10.1083/jcb.201208152 PMC3514785

[6] Parenti G., Andria G. and Ballabio A. (2015) Lysosomal storage diseases: from pathophysiology to therapy. Annu. Rev. Med. 66, 471–486 25587658 10.1146/annurev-med-122313-085916

[7] Boustany R.M. (2013) Lysosomal storage diseases–the horizon expands. Nat. Rev. Neurol. 9, 583–598 23938739 10.1038/nrneurol.2013.163

[8] Mizushima N., Levine B., Cuervo A.M. and Klionsky D.J. (2008) Autophagy fights disease through cellular self-digestion. Nature 451, 1069–1075 18305538 10.1038/nature06639 PMC2670399

[9] Ravikumar B., Sarkar S., Davies J.E., Futter M., Garcia-Arencibia M., Green-Thompson Z.W. (2010) Regulation of mammalian autophagy in physiology and pathophysiology. Physiol. Rev. 90, 1383–1435 20959619 10.1152/physrev.00030.2009

[10] Levine B. and Kroemer G. (2008) Autophagy in the pathogenesis of disease. Cell 132, 27–42 18191218 10.1016/j.cell.2007.12.018 PMC2696814

[11] Stolz A., Ernst A. and Dikic I. (2014) Cargo recognition and trafficking in selective autophagy. Nat. Cell Biol. 16, 495–501 24875736 10.1038/ncb2979

[12] Boya P., Reggiori F. and Codogno P. (2013) Emerging regulation and functions of autophagy. Nat. Cell Biol. 15, 713–720 23817233 10.1038/ncb2788 PMC7097732

[13] Zhou J., Tan S.H., Nicolas V., Bauvy C., Yang N.D., Zhang J. (2013) Activation of lysosomal function in the course of autophagy via mTORC1 suppression and autophagosome-lysosome fusion. Cell Res. 23, 508–523 23337583 10.1038/cr.2013.11 PMC3616426

[14] Lieberman A.P., Puertollano R., Raben N., Slaugenhaupt S., Walkley S.U. and Ballabio A. (2012) Autophagy in lysosomal storage disorders. Autophagy 8, 719–730 22647656 10.4161/auto.19469 PMC3378416

[15] Ward C., Martinez-Lopez N., Otten E.G., Carroll B., Maetzel D., Singh R. (2016) Autophagy, lipophagy and lysosomal lipid storage disorders. Biochim. Biophys. Acta 1861, 269–284 26778751 10.1016/j.bbalip.2016.01.006

[16] Klionsky D.J. and Schulman B.A. (2014) Dynamic regulation of macroautophagy by distinctive ubiquitin-like proteins. Nat. Struct. Mol. Biol. 21, 336–345 24699082 10.1038/nsmb.2787 PMC4036234

[17] Mizushima N., Noda T., Yoshimori T., Tanaka Y., Ishii T., George M.D. (1998) A protein conjugation system essential for autophagy. Nature 395, 395–398 9759731 10.1038/26506

[18] Kabeya Y., Mizushima N., Ueno T., Yamamoto A., Kirisako T., Noda T. (2000) LC3, a mammalian homologue of yeast Apg8p, is localized in autophagosome membranes after processing. EMBO 19, 5720–5728 10.1093/emboj/19.21.5720 PMC305793 11060023

[19] Mizushima N. (2007) Autophagy: process and function. Genes Dev. 21, 2861–2873 18006683 10.1101/gad.1599207

[20] Ganley I.G. (2013) Autophagosome maturation and lysosomal fusion. Essays Biochem. 55, 65–78 24070472 10.1042/bse0550065

[21] Saftig P. and Klumperman J. (2009) Lysosome biogenesis and lysosomal membrane proteins: trafficking meets function. Nat. Rev. Mol. Cell Biol. 10, 623–635 19672277 10.1038/nrm2745

[22] Reggiori F. and Ungermann C. (2017) Autophagosome maturation and fusion. J. Mol. Biol. 429, 486–496 28077293 10.1016/j.jmb.2017.01.002

[23] Levine B., Liu R., Dong X. and Zhong Q. (2015) Beclin orthologs: integrative hubs of cell signaling, membrane trafficking, and physiology. Trends Cell Biol. 25, 533–544 26071895 10.1016/j.tcb.2015.05.004 PMC4554927

[24] Nguyen T.N., Padman B.S., Usher J., Oorschot V., Ramm G. and Lazarou M. (2016) Atg8 family LC3/GABARAP proteins are crucial for autophagosome-lysosome fusion but not autophagosome formation during PINK1/Parkin mitophagy and starvation. J. Cell Biol. 215, 857–874 27864321 10.1083/jcb.201607039 PMC5166504

[25] McEwan D.G., Popovic D., Gubas A., Terawaki S., Suzuki H., Stadel D. (2015) PLEKHM1 regulates autophagosome-lysosome fusion through HOPS complex and LC3/GABARAP proteins. Mol. Cell 57, 39–54 25498145 10.1016/j.molcel.2014.11.006

[26] Wang Y., Li L., Hou C., Lai Y., Long J., Liu J. (2016) SNARE-mediated membrane fusion in autophagy. Semin. Cell Dev. Biol. 60, 97–104 27422330 10.1016/j.semcdb.2016.07.009 PMC5161566

[27] Itakura E., Kishi-Itakura C. and Mizushima N. (2012) The hairpin-type tail-anchored SNARE syntaxin 17 targets to autophagosomes for fusion with endosomes/lysosomes. Cell 151, 1256–1269 23217709 10.1016/j.cell.2012.11.001

[28] Diao J., Liu R., Rong Y., Zhao M., Zhang J., Lai Y. (2015) ATG14 promotes membrane tethering and fusion of autophagosomes to endolysosomes. Nature 520, 563–566 25686604 10.1038/nature14147 PMC4442024

[29] Tsuboyama K., Koyama-Honda I., Sakamaki Y., Koike M., Morishita H. and Mizushima N. (2016) The ATG conjugation systems are important for degradation of the inner autophagosomal membrane. Science 354, 1036–1041 27885029 10.1126/science.aaf6136

[30] Settembre C., Di Malta C., Polito V.A., Arencibia M.G., Vetrini F., Erdin S. (2011) TFEB links autophagy to lysosomal biogenesis. Science 332, 1429–1433 21617040 10.1126/science.1204592 PMC3638014

[31] Settembre C., Fraldi A., Medina D.L. and Ballabio A. (2013) Signals from the lysosome: a control centre for cellular clearance and energy metabolism. Nat. Rev. Mol. Cell Biol. 14, 283–296 23609508 10.1038/nrm3565 PMC4387238

[32] Medina D.L., Di Paola S., Peluso I., Armani A., De Stefani D., Venditti R. (2015) Lysosomal calcium signalling regulates autophagy through calcineurin and TFEB. Nat. Cell Biol. 17, 288–299 25720963 10.1038/ncb3114 PMC4801004

[33] He C. and Klionsky D.J. (2009) Regulation mechanisms and signaling pathways of autophagy. Annu. Rev. Genet. 43, 67–93 19653858 10.1146/annurev-genet-102808-114910 PMC2831538

[34] Sarkar S. (2013) Regulation of autophagy by mTOR-dependent and mTOR-independent pathways: autophagy dysfunction in neurodegenerative diseases and therapeutic application of autophagy enhancers. Biochem. Soc. Trans. 41, 1103–1130 24059496 10.1042/BST20130134

[35] Sarkar S., Floto R.A., Berger Z., Imarisio S., Cordenier A., Pasco M. (2005) Lithium induces autophagy by inhibiting inositol monophosphatase. J. Cell Biol. 170, 1101–1111 16186256 10.1083/jcb.200504035 PMC2171537

[36] Williams A., Sarkar S., Cuddon P., Ttofi E.K., Saiki S., Siddiqi F.H. (2008) Novel targets for Huntington’s disease in an mTOR-independent autophagy pathway. Nat. Chem. Biol. 4, 295–305 18391949 10.1038/nchembio.79 PMC2635566

[37] Kim Y.C. and Guan K.L. (2015) mTOR: a pharmacologic target for autophagy regulation. J. Clin. Invest. 125, 25–32 25654547 10.1172/JCI73939 PMC4382265

[38] Saxton R.A. and Sabatini D.M. (2017) mTOR signaling in growth, metabolism, and disease. Cell 168, 960–976 28283069 10.1016/j.cell.2017.02.004 PMC5394987

[39] Sarkar S. (2013) Chemical screening platforms for autophagy drug discovery to identify therapeutic candidates for Huntington’s disease and other neurodegenerative disorders. Drug Discover. Today 10, e137–e144 10.1016/j.ddtec.2012.09.010 24050242

[40] Levine B., Packer M. and Codogno P. (2015) Development of autophagy inducers in clinical medicine. J. Clin. Invest. 125, 14–24 25654546 10.1172/JCI73938 PMC4382267

[41] Rubinsztein D.C., Codogno P. and Levine B. (2012) Autophagy modulation as a potential therapeutic target for diverse diseases. Nat. Rev. Drug Discover. 11, 709–730 10.1038/nrd3802 PMC3518431 22935804

[42] Blommaart E.F.C., Luiken J.J.F.P., Blommaart P.J.E., van Woerkom G.M. and Meijer A.J. (1995) Phosphorylation of ribosomal protein S6 is inhibitory for autophagy in isolated rat hepatocytes. J. Biol. Chem. 270, 2320–2326 7836465 10.1074/jbc.270.5.2320

[43] Thoreen C.C., Kang S.A., Chang J.W., Liu Q., Zhang J., Gao Y. (2009) An ATP-competitive mammalian target of rapamycin inhibitor reveals rapamycin-resistant functions of mTORC1. J. Biol. Chem. 284, 8023–8032 19150980 10.1074/jbc.M900301200 PMC2658096

[44] Sarkar S., Davies J.E., Huang Z., Tunnacliffe A. and Rubinsztein D.C. (2007) Trehalose, a novel mTOR-independent autophagy enhancer, accelerates the clearance of mutant huntingtin and α-synuclein. J. Biol. Chem. 282, 5641–5652 17182613 10.1074/jbc.M609532200

[45] Jiang P. and Mizushima N. (2014) Autophagy and human diseases. Cell Res. 24, 69–79 24323045 10.1038/cr.2013.161 PMC3879707

[46] Rubinsztein D.C. (2006) The roles of intracellular protein-degradation pathways in neurodegeneration. Nature 443, 780–786 17051204 10.1038/nature05291

[47] Nixon R.A. (2013) The role of autophagy in neurodegenerative disease. Nat. Med. 19, 983–997 23921753 10.1038/nm.3232

[48] Menzies F.M., Moreau K. and Rubinsztein D.C. (2011) Protein misfolding disorders and macroautophagy. Curr. Opin. Cell Biol. 23, 190–197 21087849 10.1016/j.ceb.2010.10.010 PMC3080604

[49] Nita D.A., Mole S.E. and Minassian B.A. (2016) Neuronal ceroid lipofuscinoses. Epileptic Disord. 18, 73–88 10.1684/epd.2016.0844 27629553

[50] Palmer D.N., Barry L.A., Tyynela J. and Cooper J.D. (2013) NCL disease mechanisms. Biochim. Biophys. Acta 1832, 1882–1893 23707513 10.1016/j.bbadis.2013.05.014

[51] Mole S.E. and Cotman S.L. (2015) Genetics of the neuronal ceroid lipofuscinoses (Batten disease). Biochim. Biophys. Acta 1852, 2237–2241 26026925 10.1016/j.bbadis.2015.05.011 PMC4567481

[52] Simonati A., Pezzini F., Moro F. and Santorelli F.M. (2014) Neuronal ceroid lipofuscinosis: the increasing spectrum of an old disease. Curr. Mol. Med. 14, 1043–1051 25323868 10.2174/1566524014666141010154913

[53] Cárcel-Trullols J., Kovács A.D. and Pearce D.A. (2015) Cell biology of the NCL proteins: What they do and don’t do. Biochim. Biophys. Acta. 1852, 2242–2255 25962910 10.1016/j.bbadis.2015.04.027

[54] Brandenstein L., Schweizer M., Sedlacik J., Fiehler J. and Storch S. (2016) Lysosomal dysfunction and impaired autophagy in a novel mouse model deficient for the lysosomal membrane protein Cln7. Hum. Mol. Genet. 25, 777–791 26681805 10.1093/hmg/ddv615

[55] Thelen M., Daμμe M., Schweizer M., Hagel C., Wong A.M.S., Cooper J.D. (2012) Disruption of the autophagy-lysosome pathway is involved in neuropathology of the nclf mouse model of neuronal ceroid lipofuscinosis. PLoS One 7, e35493, 10.1371/journal.pone.0035493 22536393 PMC3335005

[56] Leinonen H., Keksa-Goldsteine V., Ragauskas S., Kohlmann P., Singh Y., Savchenko E. (2017) Retinal degeneration in a mouse model Of CLN5 disease is associated with compromised autophagy. Sci. Rep. 7, 1597 28487519 10.1038/s41598-017-01716-1 PMC5431647

[57] Cannelli N., Garavaglia B., Simonati A., Aiello C., Barzaghi C., Pezzini F. (2009) Variant late infantile ceroid lipofuscinoses associated with novel mutations in CLN6. Biochem. Biophys. Res. Commun. 379, 892–897 19135028 10.1016/j.bbrc.2008.12.159

[58] Cao Y., Espinola J.A., Fossale E., Massey A.C., Cuervo A.M., MacDonald M.E. (2006) Autophagy is disrupted in a knock-in mouse model of juvenile neuronal ceroid lipofuscinosis. J. Biol. Chem. 281, 20483–20493 16714284 10.1074/jbc.M602180200

[59] Lojewski X., Staropoli J.F., Biswas-Legrand S., Simas A.M., Haliw L., Selig M.K. (2014) Human iPSC models of neuronal ceroid lipofuscinosis capture distinct effects of TPP1 and CLN3 mutations on the endocytic pathway. Hum. Mol. Genet. 23, 2005–2022 24271013 10.1093/hmg/ddt596 PMC3959814

[60] Vidal-Donet J.M., Cárcel-Trullols J., Casanova B., Aquado C. and Knecht E. (2013) Alterations in ROS activity and lysosomal pH account for distinct patterns of macroautophagy in LINCL and JNCL fibroblasts. PLoS One 8, e55526, 10.1371/journal.pone.0055526 23408996 PMC3567113

[61] Chandrachud U., Walker M.W., Simas A.M., Heetveld S., Petcherski A., Klein M. (2015) Unbiased cell-based screening in a neuronal cell model of batten disease highlights an interaction between Ca^2+^ homeostasis, autophagy, and CLN3 Protein function. J. Biol. Chem. 290, 14361–14380 25878248 10.1074/jbc.M114.621706 PMC4505505

[62] Schultz M.L., Tecedor L., Stein C.S., Stamnes M.A. and Davidson B.L. (2014) CLN3 Deficient cells display defects in the ARF1-Cdc42 pathway and actin-dependent events. PLoS One 9, e96647, 10.1371/journal.pone.0096647 24792215 PMC4008583

[63] Shacka J.J. and Roth K.A. (2005) Cathepsin deficiency as a model for neuronal ceroid lipofuscinoses. Am. J. Pathol. 167, 1473–1476 16314462 10.1016/S0002-9440(10)61233-3 PMC1613199

[64] Koike M., Nakanishi H., Saftig P., Ezaki J., Isahara K., Ohsawa Y. (2000) Cathepsin D deficiency induces lysosomal storage with ceroid lipofuscin in mouse CNS neurons. J. Neurosci. 20, 6898–6906 10995834 10.1523/JNEUROSCI.20-18-06898.2000 PMC6772823

[65] Koike M., Shibata M., Waguri S., Yoshimura K., Tanida I., Kominami E. (2005) Participation of autophagy in storage of lysosomes in neurons from mouse models of neuronal ceroid-lipofuscinoses (Batten disease). Am. J. Pathol. 167, 1713–1728 16314482 10.1016/S0002-9440(10)61253-9 PMC1613187

[66] Shacka J.J., Klocke B.J., Young C., Shibata M., Olney J.W., Uchiyama Y. (2007) Cathepsin D deficiency induces persistent neurodegeneration in the absence of Bax-dependent apoptosis. J. Neurosci. 27, 2081–2090 17314303 10.1523/JNEUROSCI.5577-06.2007 PMC6673541

[67] Olsen A.S.B. and Færgeman N.J. (2017) Sphingolipids: membrane microdomains in brain development, function and neurological diseases. Open Biol. 7, 10.1098/rsob.170069 PMC5451547 28566300

[68] Platt F.M. (2014) Sphingolipid lysosomal storage disorders. Nature 510, 68–75 24899306 10.1038/nature13476

[69] Sandhoff K. and Kolter T. (2003) Biosynthesis and degradation of mammalian glycosphingolipids. Philos. Trans. R. Soc. B Biol. Sci. 358, 847–861 10.1098/rstb.2003.1265 PMC1693173 12803917

[70] Schuchman E.H. and Desnick R.J. (2017) Types A and B Niemann-Pick disease. Mol. Genet. Metab. 120, 27–33 28164782 10.1016/j.ymgme.2016.12.008 PMC5347465

[71] Vanier M.T. (2010) Niemann-Pick disease type C. Orphanet J. Rare Dis. 5, 16 20525256 10.1186/1750-1172-5-16 PMC2902432

[72] Storch J. and Xu Z. (2009) Niemann–Pick C2 (NPC2) and intracellular cholesterol trafficking. Biochim. Biophys. Acta 1791, 671–678 19232397 10.1016/j.bbalip.2009.02.001 PMC4281484

[73] Sarkar S., Carroll B., Buganim Y., Maetzel D., Ng A.H.M., Cassady J.P. (2013) Impaired autophagy in the lipid-storage disorder Niemann-Pick Type C1 disease. Cell Rep. 5, 1302–1315 24290752 10.1016/j.celrep.2013.10.042 PMC3957429

[74] Maetzel D., Sarkar S., Wang H., Abi-Mosleh L., Xu P., Cheng A.W. (2014) Genetic and chemical correction of cholesterol accumulation and impaired autophagy in hepatic and neural cells derived from Niemann-Pick Type C patient-specific iPS cells. Stem Cell Rep. 2, 866–880 10.1016/j.stemcr.2014.03.014 PMC4050353 24936472

[75] Boland B., Smith D.A., Mooney D., Jung S.S., Walsh D.M. and Platt F.M. (2010) Macroautophagy is not directly involved in the metabolism of amyloid precursor protein. J. Biol. Chem. 285, 37415–37426 20864542 10.1074/jbc.M110.186411 PMC2988347

[76] Liao G., Yao Y., Liu J., Yu Z., Cheung S., Xie A. (2007) Cholesterol accumulation is associated with lysosomal dysfunction and autophagic stress in Npc1^−^ ^/-^ mouse brain. Am. J. Pathol. 171, 962–975 17631520 10.2353/ajpath.2007.070052 PMC1959498

[77] Pacheco C.D., Kunkel R. and Lieberman A.P. (2007) Autophagy in Niemann-Pick C disease is dependent upon Beclin-1 and responsive to lipid trafficking defects. Hum. Mol. Genet. 16, 1495–1503 17468177 10.1093/hmg/ddm100

[78] Meske V., Erz J., Priesnitz T. and Ohm T.G. (2014) The autophagic defect in Niemann-Pick disease type C neurons differs from somatic cells and reduces neuronal viability. Neurobiol. Dis. 64, 88–97 24412309 10.1016/j.nbd.2013.12.018

[79] Ordonez M.P., Roberts E.A., Kidwell C.U., Yuan S.H., Plaisted W.C. and Goldstein L.S. (2012) Disruption and therapeutic rescue of autophagy in a human neuronal model of Niemann Pick type C1. Hum. Mol. Genet. 21, 2651–2662 22437840 10.1093/hmg/dds090 PMC3363339

[80] Lee H., Lee J.K., Park M.H., Hong Y.R., Marti H.H., Kim H. (2014) Pathological roles of the VEGF/SphK pathway in Niemann-Pick type C neurons. Nat. Commun. 5, 5514 25417698 10.1038/ncomms6514 PMC4263144

[81] Fraldi A., Annunziata F., Lombardi A., Kaiser H.J., Medina D.L., Spampanato C. (2010) Lysosomal fusion and SNARE function are impaired by cholesterol accumulation in lysosomal storage disorders. EMBO 29, 3607–3620 10.1038/emboj.2010.237 PMC2982760 20871593

[82] Lloyd-Evans E., Morgan A.J., He X., Smith D.A., Elliot-Smith E., Sillence D.J. (2008) Niemann-Pick disease type C1 is a sphingosine storage disease that causes deregulation of lysosomal calcium. Nat. Med. 14, 1247–1255 18953351 10.1038/nm.1876

[83] Kiselyov K., Yamaguchi S., Lyons C.W. and Muallem S. (2010) Aberrant Ca^2+^ handling in lysosomal storage disorders. Cell Calcium 47, 103–111 20053447 10.1016/j.ceca.2009.12.007 PMC2838446

[84] Guo H., Zhao M., Qiu X., Deis J.A., Huang H., Tang Q.Q. (2016) Niemann-Pick type C2 deficiency impairs autophagy-lysosomal activity, mitochondrial function, and TLR signaling in adipocytes. J. Lipid Res. 57, 1644–1658 27402802 10.1194/jlr.M066522 PMC5003158

[85] Singh R., Kaushik S., Wang Y., Xiang Y., Novak I., Komatsu M. (2009) Autophagy regulates lipid metabolism. Nature 458, 1131–1135 19339967 10.1038/nature07976 PMC2676208

[86] Mistry P.K., Lopez G., Schiffmann R., Barton N.W., Weinreb N.J. and Sidransky E. (2017) Gaucher disease: progress and ongoing challenges. Mol. Genet. Metab. 120, 8–21 27916601 10.1016/j.ymgme.2016.11.006 PMC5425955

[87] Hruska K.S., LaMarca M.E., Scott C.R. and Sidransky E. (2008) Gaucher disease: mutation and polymorphism spectrum in the glucocerebrosidase gene (*GBA*). Hum. Mutat. 29, 567–583 18338393 10.1002/humu.20676

[88] Wong K., Sidransky E., Verma A., Mixon T., Sandberg G.D., Wakefield L.K. (2004) Neuropathology provides clues to the pathophysiology of Gaucher disease. Mol. Genet. Metab. 82, 192–207 15234332 10.1016/j.ymgme.2004.04.011

[89] Vaccaro A.M., Motta M., Tatti M., Scarpa S., Masuelli L., Bhat M. (2010) Saposin C mutations in Gaucher disease patients resulting in lysosomal lipid accumulation, saposin C deficiency, but normal prosaposin processing and sorting. Hum. Mol. Genet. 19, 2987–2997 20484222 10.1093/hmg/ddq204

[90] Tamargo R.J., Velayati A., Goldin E. and Sidransky E. (2012) The role of saposin C in Gaucher disease. Mol. Genet. Metab. 106, 257–263 22652185 10.1016/j.ymgme.2012.04.024 PMC3534739

[91] Awad O., Sarkar C., Panicker L.M., Miller D., Zeng X., Sgambato J.A. (2015) Altered TFEB-mediated lysosomal biogenesis in Gaucher disease iPSC-derived neuronal cells. Hum. Mol. Genet. 24, 5775–5788 26220978 10.1093/hmg/ddv297

[92] Tatti M., Motta M., Di Bartolomeo S., Scarpa S., Cianfanelli V., Cecconi F. (2012) Reduced cathepsins B and D cause impaired autophagic degradation that can be almost completely restored by overexpression of these two proteases in Sap C-deficient fibroblasts. Hum. Mol. Genet. 21, 5159–5173 22949512 10.1093/hmg/dds367

[93] Sun Y., Liou B., Ran H., Skelton M.R., Williams M.T., Vorhees C.V. (2010) Neuronopathic Gaucher disease in the mouse: viable combined selective saposin C deficiency and mutant glucocerebrosidase (V394L) mice with glucosylsphingosine and glucosylceramide accumulation and progressive neurological deficits. Hum. Mol. Genet. 19, 1088–1097 20047948 10.1093/hmg/ddp580 PMC2830832

[94] Osellame L.D., Rahim A.A., Hargreaves I.P., Gegg M.E., Richard-Londt A., Brandner S. (2013) Mitochondria and quality control defects in a mouse model of gaucher disease—links to Parkinson’s disease. Cell Metab. 17, 941–953 23707074 10.1016/j.cmet.2013.04.014 PMC3678026

[95] Y-h Xu, K Xu, Sun Y., Liou B., Quinn B., R-h Li (2014) Multiple pathogenic proteins implicated in neuronopathic Gaucher disease mice. Hum. Mol. Genet. 23, 3943–3957 24599400 10.1093/hmg/ddu105 PMC4082362

[96] Farfel-Becker T., Vitner E.B., Kelly S.L., Bame J.R., Duan J., Shinder V. (2014) Neuronal accumulation of glucosylceramide in a mouse model of neuronopathic Gaucher disease leads to neurodegeneration. Hum. Mol. Genet. 23, 843–854 24064337 10.1093/hmg/ddt468 PMC3900102

[97] Kinghorn K.J., Grönke S., Castillo-Quan J.I., Woodling N.S., Li L., Sirka E. (2016) A *Drosophila* model of neuronopathic gaucher disease demonstrates lysosomal-autophagic defects and altered mTOR Signalling and is functionally rescued by rapamycin. J. Neurosci. 36, 11654–11670 27852774 10.1523/JNEUROSCI.4527-15.2016 PMC5125225

[98] Bassi M.T., Manzoni M., Monti E., Pizzo M.T., Ballabio A. and Borsani G. (2000) Cloning of the gene encoding a novel integral membrane protein, mucolipidin—and identification of the two major founder mutations causing mucolipidosis Type IV. Am. J. Hum. Genet. 67, 1110–1120 11013137 10.1016/s0002-9297(07)62941-3 PMC1288553

[99] Bargal R., Avidan N., Ben-Asher E., Olender Z., Zeigler M., Frumkin A. (2000) Identification of the gene causing mucolipidosis type IV. Nat. Genet. 26, 118–123 10973263 10.1038/79095

[100] LaPlante J.M., Ye C.P., Quinn S.J., Goldin E., Brown E.M., Slaugenhaupt S.A. (2004) Functional links between mucolipin-1 and Ca^2+^-dependent membrane trafficking in mucolipidosis IV. Biochem. Biophys. Res. Commun. 322, 1384–1391 15336987 10.1016/j.bbrc.2004.08.045

[101] Eichelsdoerfer J.L., Evans J.A., Slaugenhaupt S.A. and Cuajungco M.P. (2010) Zinc dyshomeostasis is linked with the loss of mucolipidosis IV-associated TRPML1 ion channel. J. Biol. Chem. 285, 34304–34308 20864526 10.1074/jbc.C110.165480 PMC2966043

[102] Dong X.-P., Cheng X., Mills E., Delling M., Wang F., Kurz T. (2008) The type IV mucolipidosis-associated protein TRPML1 is an endolysosomal iron release channel. Nature 455, 992–996 18794901 10.1038/nature07311 PMC4301259

[103] De Leo M.G., Staiano L., Vicinanza M., Luciani A., Carissimo A., Mutarelli M. (2016) Autophagosome-lysosome fusion triggers a lysosomal response mediated by TLR9 and controlled by OCRL. Nat. Cell Biol. 18, 839–850 27398910 10.1038/ncb3386 PMC5040511

[104] Micsenyi M.C., Dobrenis K., Stephney G., Pickel J., Vanier M.T., Slaugenhaupt S.A. (2009) Neuropathology of the *Mcoln1* ^−/−^ knockout mouse model of mucolipidosis Type IV. J. Neuropathol. Exp. Neurol. 68, 125–135 19151629 10.1097/NEN.0b013e3181942cf0 PMC4232971

[105] Venkatachalam K., Long A.A., Elsaesser R., Nikolaeva D., Broadie K. and Montell C. (2008) Motor deficit in a *Drosophila* model of mucolipidosis Type IV due to defective clearance of apoptotic cells. Cell 135, 838–851 19041749 10.1016/j.cell.2008.09.041 PMC2649760

[106] Miedel M.T., Rbaibi Y., Guerriero C.J., Colletti G., Weixel K.M., Weisz O.A. (2008) Membrane traffic and turnover in TRP-ML1–deficient cells: a revised model for mucolipidosis type IV pathogenesis. J. Exp. Med. 205, 1477–1490 18504305 10.1084/jem.20072194 PMC2413042

[107] Thompson E.G., Schaheen L., Dang H. and Fares H. (2007) Lysosomal trafficking functions of mucolipin-1 in murine macrophages. BMC Cell Biol. 8, 54, 10.1186/1471-2121-8-54 18154673 PMC2254603

[108] Curcio-Morelli C., Charles F.A., Micsenyi M.C., Cao Y., Venugopal B., Browning M.F. (2010) Macroautophagy is defective in mucolipin-1-deficient mouse neurons. Neurobiol. Dis. 40, 370–377 20600908 10.1016/j.nbd.2010.06.010 PMC4392647

[109] Venugopal B., Mesires N.T., Kennedy J.C., Curcio-Morelli C., LaPlante J.M., Dice J.F. (2009) Chaperone-mediated autophagy is defective in mucolipidosis type IV. J. Cell. Physiol. 219, 344–353 19117012 10.1002/jcp.21676

[110] Oldfors A. and DiMauro S. (2013) New insights in the field of muscle glycogenoses. Curr. Opin. Neurol. 26, 544–553 23995275 10.1097/WCO.0b013e328364dbdc

[111] Godfrey R. and Quinlivan R. (2016) Skeletal muscle disorders of glycogenolysis and glycolysis. Nat. Rev. Neurol. 12, 393–402 27231184 10.1038/nrneurol.2016.75

[112] Nishino I. (2003) Autophagic vacuolar myopathies. Curr. Neurol. Neurosci. Rep. 3, 64–69 12507414 10.1007/s11910-003-0040-y

[113] Malicdan M.C. and Nishino I. (2012) Autophagy in lysosomal myopathies. Brain Pathol. 22, 82–88 22150923 10.1111/j.1750-3639.2011.00543.x PMC8029060

[114] Raben N., Plotz P. and Byrne B.J. (2002) Acid α-glucosidase deficiency (glycogenosis Type II, Pompe disease). Curr. Mol. Med. 2, 145–166 11949932 10.2174/1566524024605789

[115] Dasouki M., Jawdat O., Almadhoun O., Pasnoor M., McVey A.L., Abuzinadah A. (2014) Pompe disease: literature review and case series. Neurol. Clin. 32, 751–776, ix 25037089 10.1016/j.ncl.2014.04.010 PMC4311397

[116] Fukuda T., Ewan L., Bauer M., Mattaliano R.J., Zaal K., Ralston E. (2006) Dysfunction of endocytic and autophagic pathways in a lysosomal storage disease. Ann. Neurol. 59, 700–708 16532490 10.1002/ana.20807

[117] Nascimbeni A.C., Fanin M., Masiero E., Angelini C. and Sandri M. (2012) The role of autophagy in the pathogenesis of glycogen storage disease type II (GSDII). Cell Death Differ. 19, 1698–1708 22595755 10.1038/cdd.2012.52 PMC3438501

[118] Raben N., Hill V., Shea L., Takikita S., Baum R., Mizushima N. (2008) Suppression of autophagy in skeletal muscle uncovers the accumulation of ubiquitinated proteins and their potential role in muscle damage in Pompe disease. Hum. Mol. Genet. 17, 3897–3908 18782848 10.1093/hmg/ddn292 PMC2638578

[119] Zirin J., Nieuwenhuis J. and Perrimon N. (2013) Role of autophagy in glycogen breakdown and its relevance to chloroquine myopathy. PLoS Biol. 11, e1001708, 10.1371/journal.pbio.1001708 24265594 PMC3825659

[120] Kotoulas O.B., Kalamidas S.A. and Kondomerkos D.J. (2004) Glycogen autophagy. Microsc. Res. Tech. 64, 10–20 15287014 10.1002/jemt.20046

[121] Nishino I., Fu J., Tanji K., Yamada T., Shimojo S., Koori T. (2000) Primary LAMP-2 deficiency causes X-linked vacuolar cardiomyopathy and myopathy (Danon disease). Nature 406, 906–910 10972294 10.1038/35022604

[122] Bandyopadhyay U., Kaushik S., Varticovski L. and Cuervo A.M. (2008) The chaperone-mediated autophagy receptor organizes in dynamic protein complexes at the lysosomal membrane. Mol. Cell. Biol. 28, 5747–5763 18644871 10.1128/MCB.02070-07 PMC2546938

[123] Tanaka Y., Guhde G., Suter A., Eskelinen E.-L., Hartmann D., Lullmann-Rauch R. (2000) Accumulation of autophagic vacuoles and cardiomyopathy in LAMP-2-deficient mice. Nature 406, 902–906 10972293 10.1038/35022595

[124] Eskelinen E.-L., Illert A.L., Tanaka Y., Schwarzmann G., Blanz J., von Figura K. (2002) Role of LAMP-2 in lysosome biogenesis and autophagy. Mol. Biol. Cell 13, 3355–3368 12221139 10.1091/mbc.E02-02-0114 PMC124165

[125] Nascimbeni A.C., Fanin M., Angelini C. and Sandri M. (2017) Autophagy dysregulation in Danon disease. Cell Death Dis. 8, e2565 10.1038/cddis.2016.475 PMC5386379 28102838

[126] Hashem S.I., Murphy A.N., Divakaruni A.S., Klos M.L., Nelson B.C., Gault E.C. (2017) Impaired mitophagy facilitates mitochondrial damage in Danon disease. J. Mol. Cell Cardiol. 108, 86–94 28526246 10.1016/j.yjmcc.2017.05.007

[127] Dowling J.J., Moore S.A., Kalimo H. and Minassian B.A. (2015) X-linked myopathy with excessive autophagy: a failure of self-eating. Acta Neuropathol. (Berl.) 129, 383–390 25644398 10.1007/s00401-015-1393-4

[128] Ramachandran N., Munteanu I., Wang P., Ruggieri A., Rilstone J.J., Israelian N. (2013) VMA21 deficiency prevents vacuolar ATPase assembly and causes autophagic vacuolar myopathy. Acta Neuropathol. (Berl.) 125, 439–457 23315026 10.1007/s00401-012-1073-6

[129] Munteanu I., Kalimo H., Saraste A., Nishino I. and Minassian B.A. (2017) Cardiac autophagic vacuolation in severe X-linked myopathy with excessive autophagy. Neuromuscul. Disord. 27, 185–187 27916343 10.1016/j.nmd.2016.10.007

[130] Mariño G., Madeo F. and Kroemer G. (2011) Autophagy for tissue homeostasis and neuroprotection. Curr. Opin. Cell Biol. 23, 198–206 21030235 10.1016/j.ceb.2010.10.001

[131] Komatsu M., Waguri S., Chiba T., Murata S., Iwata J., Tanida I. (2006) Loss of autophagy in the central nervous system causes neurodegeneration in mice. Nature 441, 880–884 16625205 10.1038/nature04723

[132] Hara T., Nakamura K., Matsui M., Yamamoto A., Nakahara Y., Suzuki-Migishima R. (2006) Suppression of basal autophagy in neural cells causes neurodegenerative disease in mice. Nature 441, 885–889 16625204 10.1038/nature04724

[133] Singh R. and Cuervo A.M. (2012) Lipophagy: connecting autophagy and lipid metabolism. Int. J. Cell Biol. 2012, 282041 22536247 10.1155/2012/282041 PMC3320019

[134] Youle R.J. and Narendra D.P. (2011) Mechanisms of mitophagy. Nat. Rev. Mol. Cell Biol. 12, 9–14 21179058 10.1038/nrm3028 PMC4780047

[135] Green D.R. and Levine B. (2014) To be or not to be? how selective autophagy and cell death govern cell fate Cell 157, 65–75 24679527 10.1016/j.cell.2014.02.049 PMC4020175

[136] Schneider J.L. and Cuervo A.M. (2014) Autophagy and human disease: emerging themes. Curr. Opin. Genet. Dev. 26, 16–23 24907664 10.1016/j.gde.2014.04.003 PMC4253630

[137] Lee J., Giordano S. and Zhang J. (2012) Autophagy, mitochondria and oxidative stress: cross-talk and redox signalling. Biochem. J. 441, 523–540 22187934 10.1042/BJ20111451 PMC3258656

[138] Sarkar S., Ravikumar B., Floto R.A. and Rubinsztein D.C. (2009) Rapamycin and mTOR-independent autophagy inducers ameliorate toxicity of polyglutamine-expanded huntingtin and related proteinopathies. Cell Death Differ. 16, 46–56 18636076 10.1038/cdd.2008.110

[139] Sarkar S., Perlstein E.O., Imarisio S., Pineau S., Cordenier A., Maglathlin R.L. (2007) Small molecules enhance autophagy and reduce toxicity in Huntington’s disease models. Nat. Chem. Biol. 3, 331–338 17486044 10.1038/nchembio883 PMC2635561

[140] Madeo F., Zimmermann A., Maiuri M.C. and Kroemer G. (2015) Essential role for autophagy in life span extension. J. Clin. Invest. 125, 85–93 25654554 10.1172/JCI73946 PMC4382258

[141] Morselli E., Galluzzi L., Kepp O., Criollo A., Maiuri M.C., Tavernarakis N. (2009) Autophagy mediates pharmacological lifespan extension by spermidine and resveratrol. Aging 1, 961–970 20157579 10.18632/aging.100110 PMC2815753

[142] Kuo S.Y., Castoreno A.B., Aldrich L.N., Lassen K.G., Goel G., Dančík V. (2015) Small-molecule enhancers of autophagy modulate cellular disease phenotypes suggested by human genetics. Proc. Natl Acad. Sci. U.S.A. 112, E4281–E4287 26195741 10.1073/pnas.1512289112 PMC4534235

[143] Sarkar S., Maetzel D., Korolchuk V.I. and Jaenisch R. (2014) Restarting stalled autophagy a potential therapeutic approach for the lipid storage disorder, Niemann-Pick type C1 disease. Autophagy 10, 1137–1140 24879158 10.4161/auto.28623 PMC4091173

[144] Palmieri M., Pal R., Nelvagal H.R., Lotfi P., Stinnett G.R., Seymour M.L. (2017) mTORC1-independent TFEB activation via Akt inhibition promotes cellular clearance in neurodegenerative storage diseases. Nat. Commun. 8, 14338 28165011 10.1038/ncomms14338 PMC5303831

[145] DeBosch B.J., Heitmeier M.R., Mayer A.L., Higgins C.B., Crowley J.R., Kraft T.E. (2016) Trehalose inhibits solute carrier 2A (SLC2A) proteins to induce autophagy and prevent hepatic steatosis. Sci. Signal. 9, ra21 26905426 10.1126/scisignal.aac5472 PMC4816640

[146] Chang J.-W., Choi H., Cotman S.L. and Jung Y.-K. (2011) Lithium rescues the impaired autophagy process in Cb*Cln3* ^Δex7/8/Δex7/8^ cerebellar cells and reduces neuronal vulnerability to cell death via IMPase inhibition. J. Neurochem. 116, 659–668 21175620 10.1111/j.1471-4159.2010.07158.x PMC4517618

[147] Lim J.-A., Li L., Shirihai O.S., Trudeau K.M., Puertollano R. and Raben N. (2017) Modulation of mTOR signaling as a strategy for the treatment of Pompe disease. EMBO Mol. Med. 9, 353–370 28130275 10.15252/emmm.201606547 PMC5331267

[148] Canonico B., Cesarini E., Salucci S., Luchetti F., Falcieri E., Di Sario G. (2016) Defective autophagy, mitochondrial clearance and lipophagy in Niemann-Pick Type B lymphocytes. PLoS One 11, e0165780, 10.1371/journal.pone.0165780 27798705 PMC5087958

[149] Pallet N. and Legendre C. (2013) Adverse events associated with mTOR inhibitors. Expert Opin. Drug Saf. 12, 177–186 23252795 10.1517/14740338.2013.752814

[150] Sardiello M., Palmieri M., di Ronza A., Medina D.L., Valenza M., Gennarino V.A. (2009) A gene network regulating lysosomal biogenesis and function. Science 325, 473–477 19556463 10.1126/science.1174447

[151] Decressac M., Mattsson B., Weikop P., Lundblad M., Jakobsson J. and Björklund A. (2013) TFEB-mediated autophagy rescues midbrain dopamine neurons from α-synuclein toxicity. Proc. Natl Acad. Sci. U.S.A. 110, E1817–E1826 23610405 10.1073/pnas.1305623110 PMC3651458

[152] Martini-Stoica H., Xu Y., Ballabio A. and Zheng H. (2016) The autophagy–lysosomal pathway in neurodegeneration: a TFEB perspective. Trends Neurosci. 39, 221–234 26968346 10.1016/j.tins.2016.02.002 PMC4928589

[153] Sardiello M. (2016) Transcription factor EB: from master coordinator of lysosomal pathways to candidate therapeutic target in degenerative storage diseases. Ann. N.Y. Acad. Sci. 1371, 3–14 27299292 10.1111/nyas.13131 PMC5032832

[154] Napolitano G. and Ballabio A. (2016) TFEB at a glance. J. Cell Sci. 129, 2475–2481 27252382 10.1242/jcs.146365 PMC4958300

[155] Medina Diego L., Fraldi A., Bouche V., Annunziata F., Mansueto G., Spampanato C. (2011) Transcriptional activation of lysosomal exocytosis promotes cellular clearance. Dev. Cell 21, 421–430 21889421 10.1016/j.devcel.2011.07.016 PMC3173716

[156] Spampanato C., Feeney E., Li L., Cardone M., Lim J.-A., Annunziata F. (2013) Transcription factor EB (TFEB) is a new therapeutic target for Pompe disease. EMBO Mol. Med. 5, 691–706 23606558 10.1002/emmm.201202176 PMC3662313

[157] Sato Y., Kobayashi H., Higuchi T., Shimada Y., Ida H. and Ohashi T. (2016) TFEB overexpression promotes glycogen clearance of Pompe disease iPSC-derived skeletal muscle. Mol. Ther.–Methods Clin. Dev. 3, 16054 27556060 10.1038/mtm.2016.54 PMC4980109

[158] Settembre C., Fraldi A., Jahreiss L., Spampanato C., Venturi C., Medina D. (2008) A block of autophagy in lysosomal storage disorders. Hum. Mol. Genet. 17, 119–129 17913701 10.1093/hmg/ddm289

[159] Desnick R.J. and Schuchman E.H. (2012) Enzyme replacement therapy for lysosomal diseases: lessons from 20 years of experience and remaining challenges. Annu. Rev. Genomics Hum. Genet. 13, 307–335 22970722 10.1146/annurev-genom-090711-163739

[160] Ratko T.A., Marbella A., Godfrey S. and Aronson N. (2013) Enzyme-Replacement Therapies for Lysosomal Storage Diseases. Agency for Healthcare Research and Quality, Comparative Effectiveness Technical Briefs, Rockville (MD) Report No.: 12(13)-EHC154-EF 23390670

[161] Cox T.M. (2010) Recommendations for treating patients with Gaucher disease with emerging enzyme products. Blood Cells Mol. Dis. 44, 84–85 20022772 10.1016/j.bcmd.2009.12.001

[162] Aerts J.M.F.G., Yasothan U. and Kirkpatrick P. (2010) Velaglucerase alfa. Nat. Rev. Drug Discover. 9, 837–838 10.1038/nrd3311 21030995

[163] Hollak C.E. (2012) An evidence-based review of the potential benefits of taliglucerase alfa in the treatment of patients with Gaucher disease. Core Evidence 7, 15–20 22654679 10.2147/CE.S20201 PMC3363131

[164] McEachern K.A., Fung J., Komarnitsky S., Siegel C.S., Chuang W.-L., Hutto E. (2007) A specific and potent inhibitor of glucosylceramide synthase for substrate inhibition therapy of Gaucher disease. Mol. Genet. Metab. 91, 259–267 17509920 10.1016/j.ymgme.2007.04.001

[165] Bennett L.L. and Mohan D. (2013) Gaucher disease and its treatment options. Ann. Pharmacother. 47, 1182–1193 24259734 10.1177/1060028013500469

[166] Ripolone M., Violano R., Ronchi D., Mondello S., Nascimbeni A., Colombo I. (2017) Effects of short-to-long term enzyme replacement therapy (ERT) on skeletal muscle tissue in late onset Pompe disease (LOPD). Neuropathol. Appl. Neurobiol. doi: 10.1111/nan.12414 10.1111/nan.12414 28574618

[167] Ashe K.M., Taylor K.M., Chu Q., Meyers E., Ellis A., Jingozyan V. (2010) Inhibition of glycogen biosynthesis via mTORC1 suppression as an adjunct therapy for Pompe disease. Mol. Genet. Metab. 100, 309–315 20554235 10.1016/j.ymgme.2010.05.001

